# Characterization and genome analysis of *Escherichia* phage fBC-Eco01, isolated from wastewater in Tunisia

**DOI:** 10.1007/s00705-022-05680-8

**Published:** 2023-01-07

**Authors:** Emna Grami, Shimaa Badawy, Saija Kiljunen, Neila Saidi, Mikael Skurnik

**Affiliations:** 1 Centre de Recherches et des Technologies des Eaux (CERTE) Laboratoire Eaux, Membranes et Biotechnologies de L’Environnement (LR19CERTE04), Technopark Borj Cedria, Tunisia; 2 Faculté des Sciences de Bizerte, Université de Carthage, 7021 Jarzouna, Tunisia; 3 Department of Bacteriology and Immunology, Human Microbiome Research Program, Faculty of Medicine, University of Helsinki, Helsinki University Hospital HUSLAB, 00290 Helsinki, Finland; 4 Department of Botany and Microbiology, Faculty of Science, Damietta University, 34517 New Damietta, Egypt

## Abstract

The rise of antibiotic resistance in bacterial strains has led to vigorous exploration for alternative treatments. To this end, phage therapy has been revisited, and it is gaining increasing attention, as it may represent an efficient alternative for treating multiresistant pathogenic bacteria. Phage therapy is considered safe, and phages do not infect eukaryotic cells. There have been many studies investigating phage-host bacteria interactions and the ability of phages to target specific hosts. *Escherichia coli* is the causative agent of a multitude of infections, ranging from urinary tract infections to sepsis, with growing antibiotic resistance. In this study, we characterized the *Escherichia* phage fBC-Eco01, which was isolated from a water sample collected at Oued, Tunis. Electron microscopy showed that fBC-Eco01 phage particles have siphovirus morphology, with an icosahedral head of 61 ± 3 nm in diameter and a non-contractile tail of 94 ± 2 nm in length and 12 ± 0.9 nm in width. The genome of fBC-Eco01 is a linear double-stranded DNA of 43.466 bp with a GC content of 50.4%. Comparison to databases allowed annotation of the functions to 39 of the 78 predicted gene products. A single-step growth curve revealed that fBC-Eco01 has a latent period of 30 minutes and a burst size of 175 plaque-forming units (PFU) per infected cell. Genomic analysis indicated that fBC-Eco01 is a member of the subfamily *Guernseyvirinae*. It is most closely related to a group of phages of the genus *Kagunavirus* that infect *Enterobacter, Raoultella*, and *Escherichia* strains.

**Supplementary Information:**

The online version contains supplementary material available at 10.1007/s00705-022-05680-8.

## Introduction

*Escherichia coli*, a species of the family *Enterobacteriaceae*, includes both commensal and pathogenic bacteria, and it is perhaps the most thoroughly studied bacterial species. This Gram-negative coliform bacterium normally lives in the intestines of animals and humans. It is also found in the environment, in wastewater, vegetation, or food. Although most strains of *E. coli* are harmless and are part of the natural intestinal flora, some *E. coli* are pathogenic and are responsible for food poisoning and may cause severe disease. Specifically, 75 to 95% of urinary tract infections (UTIs) are caused by *E. coli* [23], making it the most common causative agent of UTIs worldwide. Furthermore, *E. coli* isolates are increasingly found to be resistant to antibiotics [43], and antibiotics have even been implicated in the emergence of new forms of resistant bacteria [44]. Due to the increased prevalence of multidrug-resistant *E. coli* [58], it is essential to consider alternative treatments. Bacteriophages are considered an effective alternative for clinical applications [5].

Bacteriophages (phages) are viruses that infect bacteria [24]. They specifically recognize bacterial surface structures, including proteins and polysaccharides, which they use as receptors. After injection of the phage nucleic acid into the bacterial cytoplasm, the expressed phage proteins take over the bacterial host metabolism and the phage starts reproducing itself. At the end of its life cycle, the phage lyses the bacterial cell to liberate its progeny to the environment, thereby killing the host bacterium. This property has been exploited in the application of phages for therapeutic purposes to cure bacterial infections [49, 54].

Several studies on the potential of phage therapy to treat *E. coli* infections have been conducted over the years. In 1982, Smith and Huggins demonstrated that an intramuscular dose of an anti-K1 phage was more efficient than intramuscularly administered antibiotics in curing mice with experimental infection [51]. In addition, they demonstrated the efficacy of phages in treating experimental *E. coli* diarrhea in calves, piglets, and lambs [52]. Slopek and coworkers reported that phage therapy, with a success rate of 90 %, cured cases of septicemia, some of which were caused by *E. coli* strains that did not respond to antibiotic treatment [50]. Huff and coworkers used intramuscular bacteriophage injections to treat severe *E. coli* respiratory infections in birds, showing that the treatment proved effective within a few hours after infection [25]. The response to phage therapy was also studied using an *in vitro* model system simulating a human colon infected with an *E. coli* strain. Phage showed better treatment efficacy than ciprofloxacin [11]. Additionally, mice infected with *E. coli* O157:H7 bacteria were treated with a bacteriophage cocktail, resulting in significant reduction in the amount of the pathogen present while retaining the natural balance of the microbiota, which was not the case when ampicillin was used [14]. Manufactured probiotic-phage suppositories have been shown to successfully eliminate diarrhea in calves within 48 hours and, in addition, to stimulate immune activity against pathogenic *E. coli* [1]. Likewise, several studies have shown the ability of lytic phages to cure infections by *E. coli* bacteria for which exposure to antibiotics selects for antibiotic-resistant mutants [13]. Although bacteria also easily become resistant to bacteriophages, phages are also able to change their host range and thereby circumvent bacterial resistance [54].

Due to the increasing interest in phage therapy and the generally very narrow host range of phages, it is important to isolate and characterize new phages continually in order to achieve a high enough coverage of phages of clinically relevant pathogens to meet future demand for therapeutic phages. In the present study, we isolated and characterized a lytic *Escherichia* phage, fBC-Eco01, infecting an *E. coli* strain isolated from peritoneal puncture fluid. The properties of phage fBC-Eco01 indicate that it has therapeutic potential.

## Materials and methods

### Bacterial strains and growth media

The bacterial strains used in this work are listed in Table 1. *E. coli* strain #5629 was isolated in 2013 from peritoneal puncture fluid of a human patient at the clinical microbiology laboratory of the Helsinki University Central Hospital. The bacteria were grown at 37 °C in lysogeny broth (LB) or on LB supplemented with 1.5% Bacto Agar. For bacteriophage plaque counts, double-layer plates with 0.4% agar (soft agar) in the top layer were used.

**Table 1:** Bacterial strains and host range of fBC-Eco01

Bacterial species	Strain ID	Comments	Phage susceptibility
*Escherichia coli*	#5628	Human isolate, urinary tract infection	–
	#5629	Human isolate, peritoneal exudate, serotype O2H1	+
	#5630	Human isolate, wound infection	–
	#5631	Human isolate, wound infection	–
	EPEC 2348-69		–
	AIEC LF 82		–
*Pseudomonas aeruginosa*	ATCC 2027		–
*Pseudomonas aeruginosa*	ATCC 27853		–
*Salmonella enterica*	ATCC 13314		+
*Salmonella serovar Typhimurium*	ATCC 14028		–
*Staphylococcus aureus*	ATCC 29213		–
*Bacillus subtilis*	ATCC 6633		–
*Enterococcus faecalis*	ATCC 29212		–

(+) Lytic plaques

(–) No plaques

### Phage isolation and propagation

*E. coli* phage fBC-Eco01 was isolated from a 2-L sample of wastewater collected from Oued el Bey-Soliman Tunisia. Phage isolation was performed as described [4]. Briefly, 3.0 mL of the wastewater and 1 mL of an overnight culture of *E. coli* were added to 9 mL of tryptic soy broth (TSB, Difco). The culture was incubated at 37 °C for 4–6 h to allow the enrichment of host-strain-specific phages. Then, 500 µL of chloroform was added to the culture to lyse the bacterial cells, and the lysate was centrifuged at 5000*g* for 15 min to remove cell debris. The supernatant was passed through a 0.22-µm-pore-size filter to sterilize it. The titer of host-specific phages was determined using a double-overlay assay by pipetting 5 µL drops of serially tenfold diluted lysates onto the host bacterial soft agar overlay.

Appropriate dilutions of the enriched lysate were used to plaque-purify the phages by the soft agar overlay method as described [29]. A high-titer lysate of the phage was prepared using the semi-confluent plate lysate method [47].

### Electron microscopy

A high-titer phage suspension (8.66 × 10^10^ PFU/mL) was centrifuged at full speed (16,000*g*) for 90 min at 4 °C in Eppendorf centrifuge (5415R, rotor model 3328, Enfield, NJ, USA). The phage particles were suspended in 200 µL of 0.1 M ammonium acetate. Three µL of phage suspension was transferred to a carbon-coated copper grid, and after adsorption for 60 s, the excess liquid was removed using filter paper. The grids were then stained with 2% uranyl acetate (pH 4.2) for 30 s, and the excess dye was removed using filter paper. The grids were examined using a JEOL JEM-1400 transmission electron microscope (Jeol Ltd., Tokyo, Japan) fitted with a bottom-mounted Gatan Orius SC 1000B camera (Gatan Inc., Pleasanton, CA, USA). The specimens were inspected at 80 KV beam voltage at 80,000 and 150,000× magnification at the Electron Microscopy Unit (Institute of Biotechnology, University of Helsinki-Finland, Helsinki, Finland). The dimensions of 10 phage particles were determined and used to calculate the averages and standard errors.

### Host range

The phage host range was tested against nine Gram-negative and three Gram-positive strains (Table 1) using the spot assay method as described previously [53].

### One-step growth curve

A one-step growth curve experiment was performed as described [6] to determine the latent period and the phage burst size.

### DNA extraction, sequencing, and genome analysis

Phage DNA was extracted from a phage stock with a concentration of 8.66 × 10^10^ PFU/mL using the phenol-chloroform extraction method [46]. Bacterial genomic DNA was extracted using a GenJet Genomic DNA Purification Kit (catalog no. K0721, Thermo Fisher Scientific, Vilnius, Lithuania). The DNA samples were sequenced by the 150-bp paired-end protocol on an Illumina HiSeq platform at NovoGene (UK). To assemble the phage genome sequence, 200,000 reads were selected randomly from 8,341,604 total reads and assembled using the A5-miseq integrated pipeline [12]. The presence of genome termini was evaluated using PhageTerm [19]. The assembly was verified by mapping the 8,341,604 original reads back to the genome, using Geneious Prime 2020.1.2 assembler (Biomatters Ltd.).

Genes and the functions of their gene products were predicted using Rapid Annotation using Subsystems Technology (RAST) [3]. The predicted genes were confirmed and analyzed using Geneious Prime 2020, and open reading frames (ORFs) were predicted using ORF Finder (https://www.ncbi.nlm.nih.gov/orffinder/) together with the Artemis software [7].

Genome annotation involving functions was performed by comparison of the translated products using BLASTp, and a further search for distant homologous and conserved domains was conducted using InterProScan (http://www.ebi.ac.uk/).

Transeq and HHpred [55, 65] were used together with Clustal Omega to align multiple sequences (http://www.ebi.ac.uk/Tools/msa/clustalo/).

Putative tRNA genes were identified using tRNAscan-SE [10]. Phage-RNA-polymerase-specific promoters were predicted using PHIRE ver. 1.00 [32], host-RNA-polymerase-specific promoters were predicted using Galaxy [48], terminators were predicted using ARNold http://rssf.i2bc.paris-saclay.fr [20, 34, 39], and genome comparisons with other phages were performed using TBLASTX on the DiGAlign Dynamic Genomic Alignment server (https://www.genome.jp/digalign) [40].

Raw reads of the bacterial genome were uploaded and analysed using the EnteroBase database [64] available at https://enterobase.warwick.ac.uk/ under the barcode ESC_YA7726AA.

### Phylogenetic analysis

The fBC-Eco01 genome sequence was compared with those of a number of the most closely related phages using the MEGA11 [56]. We also used ViPTree analysis, version 2.0 [39], a phage proteomic tree dendrogram program that reveals global genomic similarity relationships between viruses with sequences in the database. In this case, the tree was generated from selected best-fit genome sequences to obtain a viral proteomic tree of fBC-Eco01 with 47 sequences of double-stranded DNA (dsDNA) viruses belonging to the class *Caudoviricetes* [57] that have gammaproteobacteria as their hosts. The ViPTree analysis was performed by the maximum-likelihood (ML) method.

### Nucleotide sequence accession number

The genomic sequence of fBC-Eco01 was submitted to the GenBank database under the accession number OM272052.1.

## Results and discussion

### Phage isolation and host range

**Fig. 1:**
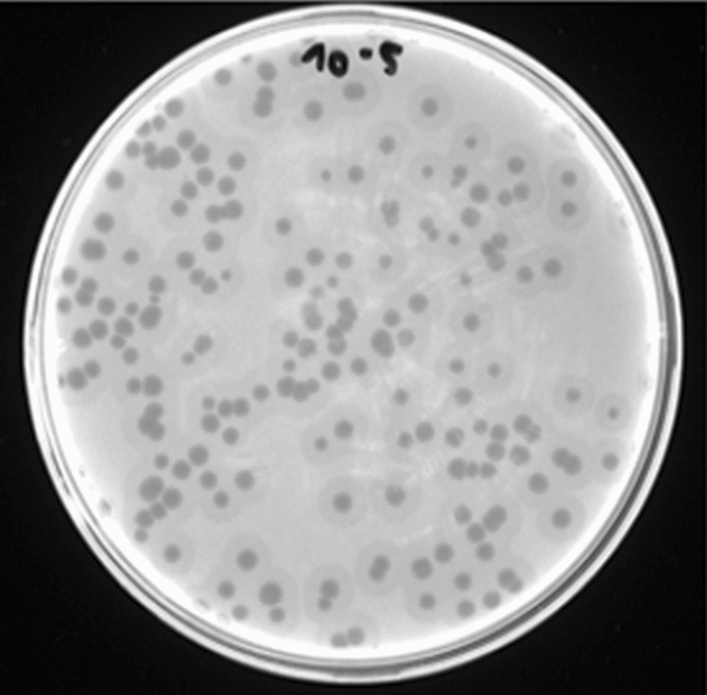
Plaques induced by phage fBC-Eco01 after 24 h of incubation at 37 °C on a lawn of *E. coli* strain #5629

A wastewater sample from Oued Borj Ecedria was screened for phages against four clinical *E. coli* human isolates (Table 1). A phage infecting strain #6529, named fBC-Eco01, was isolated. The phage formed plaques that were 1.8 ± 0.2 mm in diameter (Fig. 1). The host range of fBC-Eco01 was tested against nine bacterial strains (Table 1). Contrary to our expectations, fBC-Eco01 did not infect the other *E. coli* strains but was able to lyse *Salmonella enterica* ATCC 13314 in addition to its original *E. coli* host strain #5629. Analysis of the whole genome sequence of strain #5629 carried out using the Enterobase database indicated that the strain belongs to the pathogenic *E. coli* ST73 lineage, phylogroup B2, serotype O2:H1 [27]. The estimated genome size of this strain is 5,131,390 bp.

### Morphology analysis by TEM

**Fig. 2:**
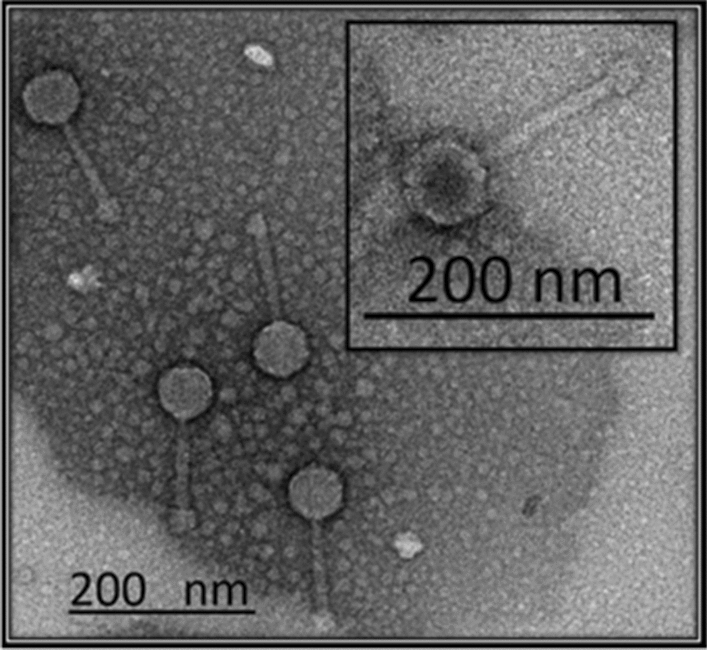
Transmission electron micrograph of phage fBC-Eco01 particles negatively stained with 2% uranyl-acetate

The morphological features of phage fBC-Eco01 particles revealed that it has an icosahedral head that is 61 ± 3 nm in diameter and a long non-contractile tail that is 94 ± 2 nm in length and 12 ± 0.9 nm in width (Fig. 2). Based on this, fBC-Eco01 is morphologically a siphovirus.

### One-step growth curve

**Fig. 3:**
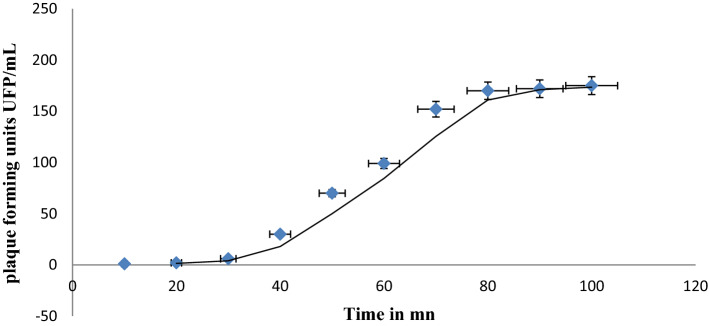
One-step growth curve of fBC-Eco01 on host *E. coli* #5629

A one-step growth curve experiment (Fig. 3) revealed that fBC-Eco01 has a latent period of about 30 min and a burst size of 175 PFU/cell with a 30 min rise phase.

### Genome analysis of *E. coli* phage fBC-Eco01

**Fig. 4:**
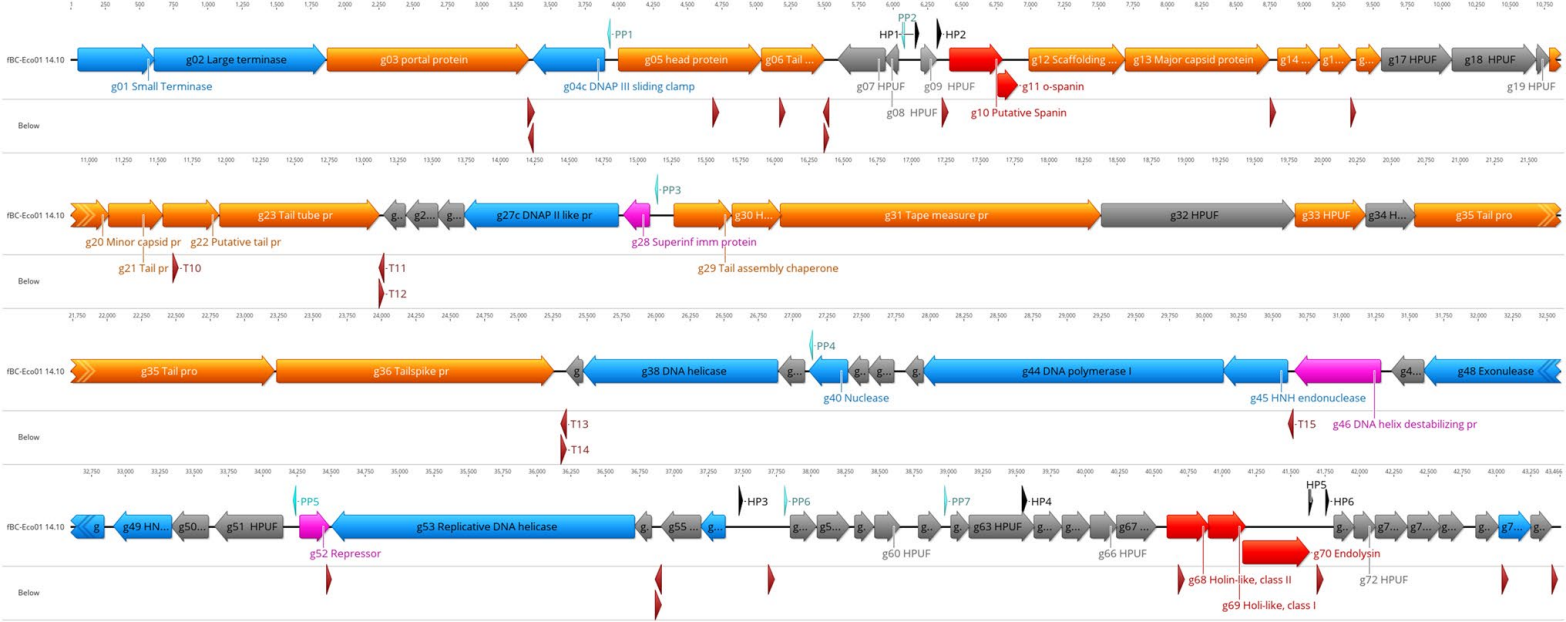
Genomic map of fBC-Eco01. The predicted genes are shown as colored arrows labeled with predicted functions (genes encoding structural proteins, orange; nucleic acid enzymes, blue; hypothetical proteins, grey; lysis functions, red; regulatory proteins, purple). For details, see Supplementary Table S1. Pr, protein; HP, host promoter (black arrowhead); PP, phage promoter (turquoise arrowhead); T, terminator (brown arrowhead); HPUF, hypothetical protein of unknown function. The map was drawn with Geneious 22.2.2 (www.geneious.com)

The fBC-Eco01 genome is a linear double-stranded DNA, 43,466 bp in length. The median depth of read coverage of the assembly was 651, and PhageTerm analysis did not identify obvious genomic termini. The genome contains 78 predicted genes, with 38 encoding proteins whose functions could be predicted based on sequence similarity to known proteins. The remaining 40 gene products were annotated as hypothetical proteins [57]. Of the predicted genes, 51 and 27 were encoded on the forward and reverse strand, respectively. The GC content of the phage is 50.4%. The overall organization of the genome of fBC-Eco01 is presented in Fig. 4, and the annotation of the predicted genes is presented in Supplementary Table S1.

Of the predicted gene products, 44 shared similarity with proteins of *E. coli* phages, and 26 gene products matched those of phages with siphovirus morphology, originating from various human metagenome projects from which partial genome sequences were submitted to the Laboratory of Cellular Oncology, National Cancer Institute, USA [57]. No tRNA genes were present in the genome. In addition, no genes encoding virulence factors, integrases, toxins, or factors associated with a lysogenic cycle were detected, suggesting that fBC-Eco01 could be used safely for therapeutic purposes.

Thirteen promoters and 23 rho-independent terminators were predicted from the sequence (Supplementary Table S2), the locations of which are indicated in Fig. 4. Of the promoters, seven were predicted to be used by the phage-encoded RNA polymerase, with the consensus sequence tgAATAGTa-ccTATTat-T, and the other six were predicted to be used by the host RNA polymerase. Most of the rho-independent terminators were located in intergenic regions, but a few were located within genes, suggesting that they might play a role in regulation of transcription.

The predicted functions of the phage gene products, based on database searches, are shown in Supplementary Table S1. Putative functions could be assigned to 37 gene products belonging to five functional groups.

### General features of the fBC-Eco01 genome

#### DNA packaging

Packaging of DNA into the phage head is carried out by the terminase complex containing the small and large terminase subunits (Gp01 and Gp02, respectively). The terminase recognizes the *cos* site of the phage genome, where it introduces nicks to generate the cohesive ends of the genome and separates them in a reaction requiring ATP hydrolysis [15]. The terminase and the phage portal proteins (Gp03) are believed to be the initiators of head assembly [8].

#### Head and tail morphogenesis genes

Fifteen gene products were predicted to be structural proteins, the genes for which are located on the forward strand of the DNA, grouped in three clusters. Gp6, Gp21, Gp22, Gp23, Gp31, Gp35, and Gp36 were predicted to be structural components of the tail. The 779-amino-acid (aa)-long Gp31 is predicted to be the tail tape-measure protein, which is the second longest gene product after the 852-aa-long tail protein Gp35. Gp31 is predicted to be involved in facilitating the phage DNA injection process in addition to determining the length of the tail [61]. A comparison of Gp30 sequences showed 99% identity to that of the kagunavirus *Escherichia* phage Schulenburg.

The predicted tail spike protein Gp35, which interacts with host receptor, is 99.92% identical to that of *Escherichia* lytic phage vB-EcoM_CBA120, a member of the newly created genus *Kuttervirus.* It is closely related to contractile-tailed phages that infect *E. coli* and *Salmonella* sp. that characteristically carry several tail fiber and tailspike proteins [22]. The nearly identical sequences of the tail spike proteins suggest that these two phages may also have a similar host range [53].

Overall, all of the tail-associated gene products, except for the tail spike protein, were very similar to their counterparts in *Escherichia* phages belonging to the genus *Kagunavirus* (94–100% identity).

Similarly, the head morphogenesis genes shared 95–99% identity with their counterparts in other *Escherichia* phages of the genus *Kagunavirus*. These include Gp05, Gp12 and Gp13, and Gp20, which are annotated as scaffold protein and major and minor capsid proteins, respectively, that initiate the formation of the procapsid [42]. In addition, Gp14 was predicted to be a head protein involved in protein interactions [28], and Gp15 was identified as a capsid decoration protein associated with stabilizing the head structure and the head-tail joining protein (Fig. 4).

#### DNA replication, recombination, and repair genes

The gene products predicted to be involved in DNA replication, recombination, and repair are encoded on the reverse strand of the DNA (indicated in the gene and gene product names by a final "c"). Gp44c was predicted to be DNA polymerase I, and Gp27c was predicted to be the DNA polymerase II small subunit, which plays a role in repairing damaged DNA by filling gaps or re-initiating DNA synthesis, as the holoenzyme is responsible for proofreading during DNA replication. Gp04c is predicted to be the clamp protein or DNA polymerase III sliding clamp, also known as a β-clamp, which binds the DNA polymerase to the DNA and prevents the enzyme from dissociating from the template DNA strand [18, 21]. Moreover, the protein–protein interactions between the clamp and the polymerase are stronger and more specific than the direct interactions between the polymerase and the template DNA strand. The DNA clamp can increase the rate of DNA synthesis up to 1,000-fold when compared to a non-processive polymerase [41].

Gp38c and Gp53c are predicted to be DNA helicases that unwind the dsDNA to allow transcription or DNA replication [31]. Gp45c and Gp49c are predicted to be HNH endonucleases that may promote the packaging process [26]. They can nick the double-stranded DNA and may play a variety of roles in replication, recombination, and repair pathways and are involved in pathogenicity [45, 60].

HNH endonucleases have also been shown to act as mobile components that are deployed through horizontal transfer and genetic exchange [38], along with Gp77, which shows similarity to the NinH protein, which affects both cellular and viral DNA processing as well as gene expression, with advantageous effects on phage multiplication and dissemination [9]. The NinH-encoding gene is located at the end of the phage genome, at the same location where the 97% identical NinH-encoding gene of *Raoultella* phage RP180 is found.

Gp40c is predicted to be a nuclease that is important for various cellular processes. Gp46c, which is predicted to be a single-strand binding protein (SSBs), may help to form the central nucleoprotein complex substrate for DNA replication, recombination, and repair processes. Finally, Gp48c, annotated as an exonuclease, is likely to be required for DNA replication and DNA repair [2, 17, 35]. It is 100% identical to its counterpart in the *Escherichia* kagunavirus VB-EcoS-Golestan [62]. Gp52c and Gp56c are predicted to possess helix-turn-helix (HTH) motifs, suggesting that they are capable of binding DNA. Each monomer contains two α-helices, joined by a short strand of amino acids, that bind to the major groove of DNA. Gp28c is predicted to be a superinfection immunity protein that is likely to be involved in gene regulation [36].

#### Lysis genes

Five gene products were predicted to play a role in the lysis process. Gp11 and Gp12, which are predicted to form the spanin complex, whose main role is to disrupt the outer membrane, share 97% and 98% identity with their counterparts in *Escherichia* phage vB-EcoS_fPoEco01 (QNO11794.1) and *Escherichia* phage phiWAO78-1, respectively. The genes encoding the other three proteins are located almost at the end of the genome. These include Gp68, a class II holin-like protein, Gp69, a class I holin-like protein, and Gp70, an endolysin. Bacterial lysis occurs when the holin generates small pores in the membrane that allow the endolysin to leak from the cytoplasm to degrade the peptidoglycan [63], resulting in release of the phage progeny to the environment [30]. Gp70 endolysin functions as the phage-encoded peptidoglycan hydrolase that breaks down the bacterial peptidoglycan at the end of the phage reproduction cycle [33]. Gp68, Gp69, and Gp70 share 79, 98, and 95% identity, respectively, with their counterparts in *Raoultella* phage RP180. Furthermore, the lysis gene organization in the fBC-Eco01 genome is similar to that of *Raoultella* phage RP180 (NC_048181) [16].

#### Phylogeny and comparative analysis

**Fig. 5:**
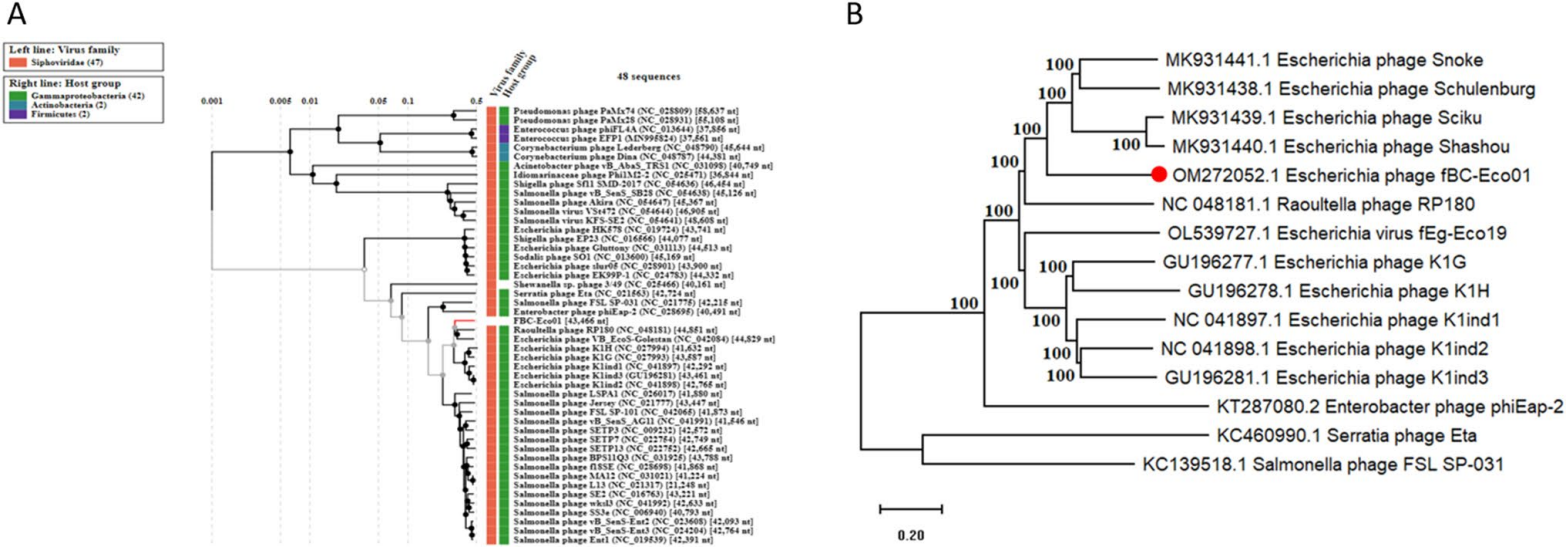
Phylogenetic analysis of fBC-Eco01. (A) Viral proteomic tree based on genomic similarity between dsDNA viruses belonging to the class *Caudoviricetes* that mainly use gammaproteobacteria as a host (Supplementary Table S3). Branch lengths are logarithmically scaled, the log scale on top represents the SG values (normalized scores by BLASTx). (B) Phylogenetic tree constructed in MEGA11 based on phage genome sequences. The GenBank accession numbers of the individual phages are indicated. The UPGMA method was used with 1000 bootstrap replicates. Bootstrap values are indicated on nodes. Phage fBC-Eco01 is indicated by a red circle

In order to determine the evolutionary position of phage fBC-Eco01, phylogenetic analysis was performed. A viral proteomic tree was constructed by the maximum-likelihood method, using the Viral Proteomic Tree server based on nucleotide sequences of 47 selected dsDNA viruses (Fig. 5A). The analysis revealed that fBC-Eco01 is closely related to *Raoultella* phage RP180 (NC_048181), with a significant genomic similarity (SG) level (> 0.6), and also to *Escherichia* phages VB-EcoS-Golestan, K1G, K1ind1, K1ind2, and K1ind3 (Supplementary Table S3). These phages belong to the genus *Kagunavirus* within the family *Guernseyvirinae*, suggesting that fBC-Eco01 is a novel member of this group. Similarity searches also showed that phage fBC-Eco01 shows considerable similarity to *Salmonella* phages of the genus *Jerseyvirus*, with an average genomic similarity of 0.5 to *Salmonella* phage LSPA (NC_026017.1) (Supplementary Table S3).

A heat map was generated that shows pairwise intergenomic distances pertaining to the nucleotide and amino acid sequence identities between the genomes of fBC-Eco-01 and phages selected based on genomic similarities (Supplementary Table S4). The heat map demonstrates that fBC-Eco01 is more closely related to kagunaviruses (74.11–78.37% sequence identity) than to jerseyviruses (39.52–71.29% sequence identity), both of which belong to the subfamily *Guernseyvirinae*. The complete genome fBC-Eco01 shares 76.89% nucleotide sequence identity with *Raoultella* phage RP180 and 71.29% identity with *Salmonella* phage LSPA1 (a jerseyvirus with a genome of 41880 bp) (Supplementary Table S4). Alignments of the genome sequences of those viruses to that of fBC-Eco01, performed using TBLASTX, are shown in Supplementary Figure S1. These comparisons indicated that phage fBC-Eco01 is most closely related to members of the genera *Kagunavirus* and *Jerseyvirus* within the subfamily *Guernseyvirinae*.

**Fig. 6:**
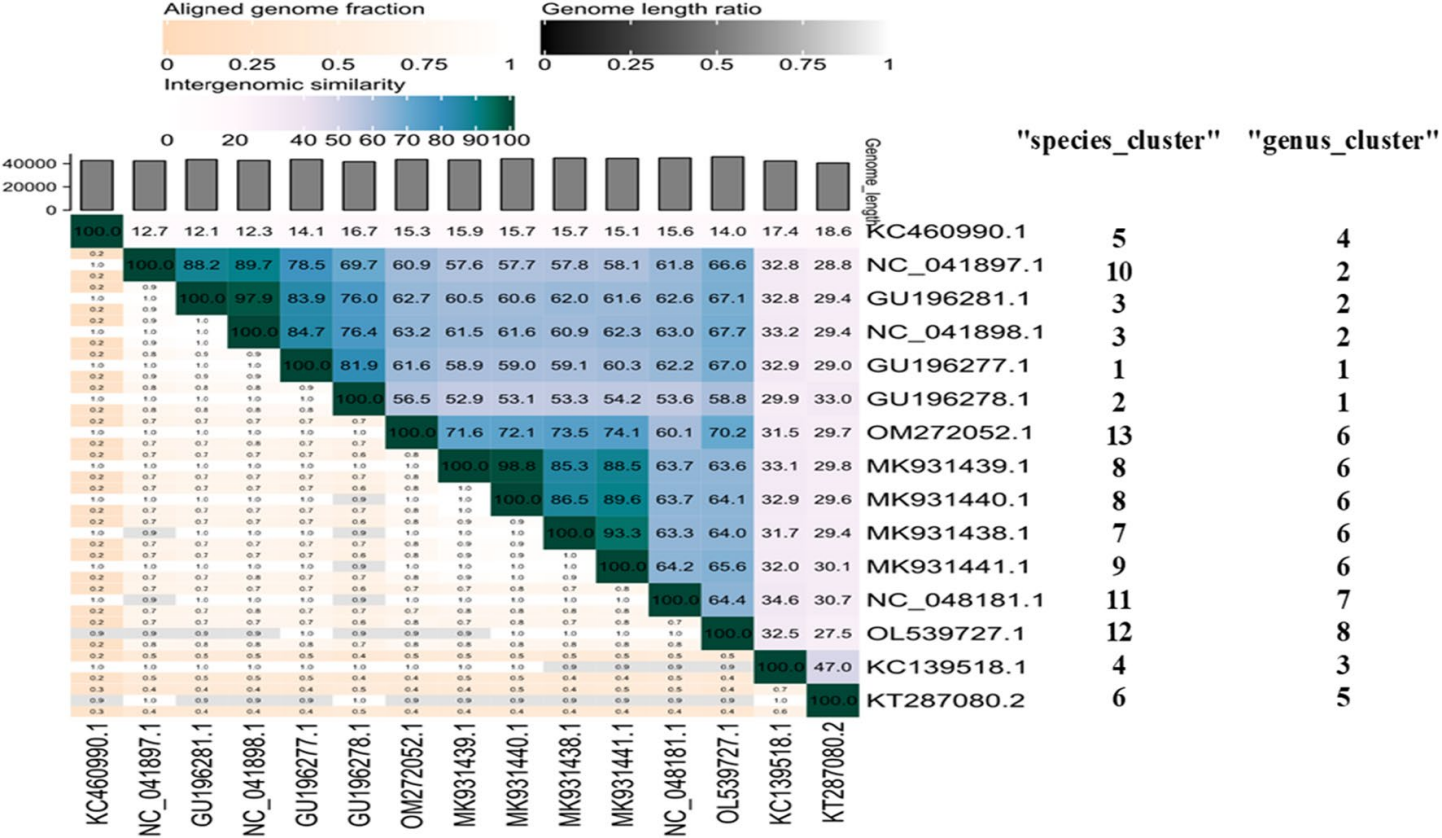
Whole-genome comparison and clustering of phage fBC-ECO01 (OM272052.1) with its closest relatives, carried out with VIRIDIC (Virus Intergenomic Distance Calculator). In the top right corner of the heat map, different shades of blue indicate intergenomic similarity (%) between the genomes of each pair compared. Numerical values are also shown. The darker the color, the more closely related are the genomes. The lower left corner of the heat map shows three indicator values for each genome pair: top value, the aligned fraction of genome 1 for the genome in this row; middle value, the genome length ratio for the two genomes in this pair; bottom value, the aligned fraction of genome 2 for the genome in this column. The darker colors represent lower values, as indicated above the heat map. The aligned genome fractions decrease with increased distance between the phages. KC460990, *Serratia* phage Eta; NC_041897, *Escherichia* phage K1ind1; GU196281, *Escherichia* phage K1ind3; NC_041898, *Escherichia* phage K1ind2; GU196277, *Escherichia* phage K1G; GU196278, *Escherichia* phage K1H; MK931438, *Escherichia* phage Schulenburg; MK931439, *Escherichia* phage Sciku; MK931440, *Escherichia* phage Shashou; MK931441, *Escherichia* phage Snoke; NC_048181, *Raoultella* phage RP180; OL539727, *Escherichia* phage fEg-Eco19; KC139518, *Salmonella* phage FSL SP-031; KT287080, *Enterobacter* phage phiEap-2

Finally, Virus Intergenomic Distance Calculator (VIRIDIC) analysis including four additional novel related phages (Sciku, Shashou, Schulenburg, and Snoke; MK931439.1, MK931440.1, MK931438.1, and MK931441.1, respectively) not present in the VipTree analysis (Fig. 5A) [37] indicated that *Escherichia* phage Snoke (MK931441.1) is the most similar (98% identity) to fBC-Eco01 (Fig. 6). A phylogenetic tree constructed in MEGA 11 based on an fBC-Eco01 alignment of whole genome sequences (Fig. 5B) confirmed that *Escherichia* phage Snoke appears to be the closest relative.

Based on the convention that a genus is formed of a cohesive group of viruses sharing more than 70% nucleotide sequence identity over the entire length of their genomes [59], we propose that phage fBC-Eco01 belongs to the genus *Kagunavirus* (https://ictv.global/taxonomy/). However, the VIRIDIC analysis would suggest that this group of phages should be re-examined with the potential of creating a new taxon (genus) for OM272052.1_fBC-Eco01, MK931439.1_Sciku, MK931440.1_Shashou, MK931438.1_Schulenburg, and MK931441.1_Snoke.

## Conclusions

A novel lytic phage, fBC-Eco01, isolated from wastewater in Tunisia, infected an *E. coli* strain of serotype O2:H1, sequence type ST73, isolated from a peritoneal puncture fluid sample from a Finnish patient. Based on electron microscopy and phylogenetic analysis, fBC-Eco01 belongs to the class *Caudoviricetes*, morphologically resembles siphoviruses, and can be assigned to the genus *Kagunavirus* of the subfamily *Guernseyvirinae*. Genome comparisons revealed that fBC-Eco01 has high sequence similarity (74–98% identity) to kagunaviruses and somewhat less (39–71% identity) to jerseyviruses. Annotation of the predicted genes revealed gene products of several functional groups, including DNA packaging, structural proteins (head and tail morphogenesis genes), DNA replication, recombination and repair genes, regulatory proteins, and host lysis proteins. Phage fBC-Eco01 has a relatively narrow host range, as it was only able to lyse *Salmonella enterica* strain ATCC 13314 besides its own isolation host, *E. coli* strain #5629.

## Supplementary Information

Below is the link to the electronic supplementary material.

Supplementary material 1 (DOCX 518.4 kb)

## Data Availability

The authors confirm that the data supporting the findings of this study are available within the article and its supplementary tables and figure.
