# Supplementary material for: Characterization and genome analysis of Escherichia phage fBC-Eco01, isolated from wastewater in Tunisia
Source: Arch Virol. 2023 Jan 7;168(2):44. doi: 10.1007/s00705-022-05680-8 (PMC9825357; doi:10.1007/s00705-022-05680-8)
Supplement: Supplementary file 1 — Supplementary material 1 (DOCX 518.4 kb) [file 705_2022_5680_MOESM1_ESM.docx]

Supplementary Tables and Figure

**Characterization and genome analysis of an *Escherichia* phage fBC-Eco01 isolated from Tunisian waste water**

Emna Grami^1,2^, Shimaa Badawy^3,4^, Saija Kiljunen^3^ ,Neila Saidi^1^ and Mikael Skurnik^3*^

^1^ Centre de Recherches et des Technologies des Eaux (CERTE) Laboratoire Eaux, Membranes et Biotechnologies de L’Environnement (LR19CERTE04), Technopark Borj Cedria, Tunisia

^2^ Faculté des Sciences de Bizerte, Université de Carthage, 7021, Tunisia,

^3^ Department of Bacteriology and Immunology, Human Microbiome Research Program, Faculty of Medicine, University of Helsinki and Helsinki University Hospital, HUSLAB

^4^ Department of Botany and Microbiology, Faculty of Science, Damietta University, New Damietta, 34517, Egypt

*Corresponding author:

Department of Bacteriology and Immunology, Human Microbiome Research Program, Faculty of Medicine, University of Helsinki.

E-mail: mikael.skurnik@helsinki.fi

Tel: +358503360981

**Supplementary Table S1.** The predicted gene products of phage fBC-Eco01 and their predicted functions

| **Gp** | **Genomic location** | **MW** | | **AA** | | **Predicted function** | | **Best hit** | | **BLASTP e-value(Identity)** | | **Query coverage %** | | **Organism** | |
| --- | --- | --- | --- | --- | --- | --- | --- | --- | --- | --- | --- | --- | --- | --- | --- |
| Gp01 | 51:611 forward | 20617 | | 186 | | Terminase small subunit | | DAQ25537.1 | | 7e-120 (99.46%) | | 100 | | Siphoviridae sp. | |
| Gp02 | 608:1858 forward | 45910 | | 417 | | Terminase large subunit | | YP_009168834.1 | | 0(99.76%) | | 100 | | *Escherichia* phage K1-dep(1) | |
| Gp03 | 1871:3340 forward | 54298 | | 490 | | Portal protein | | QEG06791.1 | | 0(97.34%) | | 100 | | *Escherichia* phage Schulenburg | |
| Gp04c | 3371:3892 reverse | 19668 | | 174 | | DNA polymerase III sliding clamp | | QNO11567.1 | | 1e-128(100%) | | 100 | | *Escherichia* phage vB_EcoS_fFiEco02 | |
| Gp05 | 3993:5036 forward | 38552 | | 348 | | Head protein | | AUE23503.1 | | 0(95.39%) | | 100 | | *Escherichia* phage vB_EcoS_HSE2 | |
| Gp06 | 5036:5497 forward | 16379 | | 154 | | Putative tail protein | | QNO11642.1 | | 9E-103 (94%) | | 100 | | *Escherichia* phage vB_EcoS_fFiEco03 | |
| Gp07c | 5594:5938 reverse | 12170 | | 115 | | Hypothetical protein FDJ09_gp05 | | YP_009620085.1 | | 2e-80(99.12%) | | 100 | | *Escherichia* phage VB_EcoS-Golestan | |
| Gp08c | 5935:6036 reverse | 3409 | | 34 | | Hypothetical protein FDJ09_gp06 | | YP_009620086.1 | | 3e-15(96.97%) | | 100 | | *Escherichia* phage VB_EcoS-Golestan | |
| Gp09 | 6200:6316 forward | 4286 | | 39 | | Hypothetical protein | | DAG81205.1 | | 7e-18 (94.74%) | | 100 | | Siphoviridae sp. | |
| Gp10 | 6411:6797 forward | 13852 | | 129 | | Putative spanin | | QNO11794.1 | | 2e-88 (96.88%) | | 100 | | *Escherichia* phage vB_EcoS_fPoEco01 | |
| Gp11 | 6760:6906 forward | 16301 | | 49 | | O-spanin | | QQV88032.1 | | 6e-25 (98%) | | 100 | | *Escherichia* phage phiWAO78-1 | |
| Gp12 | 6989:70690 forward | 25494 | | 234 | | Scaffolding protein | | QIG59215.1 | | 6e-109 (99%) | | 100 | | *Escherichia* phage vB_EcoS_XY1 | |
| Gp13 | 7693:8742 forward | 37792 | | 350 | | Major capsid protein | | ADA82453.1 | | 0(97.71%) | | 100 | | *Escherichia* phage K1-ind(3) | |
| Gp14 | 8802:9101 forward | 10042 | | 100 | | Head fiber protein | | 3QC7_A | | 2,10E-08 | | 98,73 | | *Bacillus* phage phi29 | |
| Gp15 | 9114:9341 forward | 7691 | 76 | | Capsid decoration protein | | QNO11572.1 | | 3e-34 (63.96%) | | 100 | | *Escherichia* phage vB_EcoS_fFiEco02 | |  |
| Gp16 | 9378:9557 forward | 6609 | 60 | | Head-tail joining protein | | QIG59212.1 | | 4e-36 (98.28%) | | 98 | | *Escherichia* phage vB_EcoS_XY1 | |  |
| Gp17 | 9561:10073 forward | 17680 | 171 | | Hypothetical protein FE3_012 | | QNO11650.1 | | 6e-117 (97.06%) | | 100 | | *Escherichia* phage vB_EcoS_fFiEco03 | |  |
| Gp18 | 10076:10690 forward | 20808 | 205 | | hypothetical protein Schulenburg_017 | | QEG06805.1 | | 3e-137 (95.59%) | | 100 | | *Escherichia* phage Schulenburg | |  |
| Gp19 | 10690: 10788 forward | 3500 | 33 | | Protein of unknown function (DUF3042) | | DAU29104.1 | | 5e-13 (100.00%) | | 100 | | Siphoviridae sp. | |  |
| Gp20 | 10785:11144 forward | 12917 | 120 | | Minor capsid protein | | DAU29048.1 | | 6e-80 (99.16%) | | 100 | | Siphoviridae sp | |  |
| Gp21 | 11141:11536 forward | 14507 | 132 | | Tail component | | DAU29049.1 | | 3e-94 (100.00%) | | 100 | | Siphoviridae sp. | |  |
| Gp22 | 11536:11949 forward | 14999 | 138 | | Putative tail protein | | QNO11579.1 | | 1e-97 (99.27%) | | 100 | | *Escherichia* phage vB_EcoS_fFiEco02 | |  |
| Gp23c | 11952:13118 forward | 40900 | 389 | | Tail tube protein | | DAU29051.1 | | 0.0 (97.68%) | | 100 | | Siphoviridae sp. | |  |
| Gp24c | 13147:13305 reverse | 6423 | 53 | | Hypothetical protein Schulenburg_022 | | QEG06810.1 | | 6e-31 (96.15%) | | 100 | | *Escherichia* phage Schulenburg | |  |
| Gp25c | 13313:13543 reverse | 8681 | 77 | | Hypothetical protein Schulenburg_023 | | QEG06811.1 | | 1e-50 (98.68%) | | 100 | | *Escherichia* phage Schulenburg | |  |
| Gp26c | 13533:13733 reverse | 7497 | 67 | | hypothetical protein Schulenburg_024 | | QEG06812.1 | | 1e-41 (96.97%) | | 100 | | *Escherichia* phage Schulenburg | |  |
| Gp27c | 13730:14860 reverse | 42426 | 377 | | DNA polymerase II small subunit | | DAU29064.1 | | 0.0 (100.00%) | | 100 | | Siphoviridae sp. | |  |
| Gp28 | 14899:15090 reverse | 7341 | 64 | | Superinfection immunity protein | | QEG06814.1 | | 8e-39 (100.00%) | | 100 | | *Escherichia* phage Schulenburg | |  |
| Gp29 | 15269:15685 forward | 15897 | 139 | | Hypothetical protein | | UMW87993.1 | | 1e-92 (96%) | | 100 | | *Escherichia* phage UTI-CM001 | |  |
| Gp30 | 15691:16050 forward | 13689 | 120 | | Hypothetical protein | | UMW87994.1 | | 5e-83 (99.16%) | | 100 | | *Escherichia* phage UTI-CM001 | |  |
| Gp31 | 16043:18379 forward | 83704 | 779 | | Tape measure protein | | QEG06817.1 | | 0.0 (98.97%) | | 100 | | *Escherichia* phage Schulenburg | |  |
| Gp32 | 18383:19795 forward | 50459 | 471 | | Hypothetical protein | | DAU29110.1 | | 0.0 (98.94%) | | 100 | | Siphoviridae sp. | |  |
| Gp33 | 19799:20314 forward | 18805 | 172 | | Putative baseplate protein | | 6TEH_C | | 2,90E-09 (HHPred prediction) | | 99,11 | | Rhodobacter | |  |
| Gp34 | 20311:20676 forward | 14325 | 122 | | hypothetical protein CPT_Shashou_031 | | QEA09429.1 | | 2e-88 (100.00%) | | 100 | | *Escherichia* phage Shashou | |  |
| Gp35 | 20667:23222 forward | 94289 | 852 | | Tail protein | | QNO11668.1 | | 0.0 (99%) | | 100 | | *Escherichia* phage vB_EcoS_fFiEco03 | |  |
| Gp36c | 23235:25259 forward | 71612 | 675 | | Tailspike protein | | 6NW9_A | | 2E-22 (99,92%) | | 100 | | PHAGE CBA120 | |  |
| Gp37c | 25344:25469 reverse | 4597 | 42 | | Hypothetical protein OOCIFDHN_00053 | | QQO88002.1 | | 5e-10 (68.57%) | | 85 | | *Salmonella* phage vB_SenS_ER21 | |  |
| Gp38c | 25466:26890 reverse | 53407 | 475 | | DNA helicase | | YP_008239745.1 | | 0.0 (96.62%) | | 100 | | *Salmonella* phage Jersey | |  |
| Gp39c | 26893:27087 reverse | 7250 | 65 | | Hypothetical protein L_63 | | AQN31888.1 | | 7e-40 (98.44%) | | 100 | | *Escherichia* phage L AB-2017 | |  |
| Gp40c | 27118:27402 reverse | 10802 | 95 | | Nuclease | | DAM60928.1 | | 4e-63 (95.74%) | | 100 | | Siphoviridae sp. | |  |
| Gp41c | 27399:27551 reverse | 5586 | 51 | | Hypothetical protein FE2_039 | | QNO11600.1 | | 1e-21 (92.00%) | | 100 | | *Escherichia* phage vB_EcoS_fFiEco02 | |  |
| Gp42c | 27548:27739 reverse | 7293 | 64 | | Hypothetical protein | | DAH67740.1 | | 2e-34 (84.38%) | | 100 | | Siphoviridae sp. | |  |
| Gp43c | 27822:27953 reverse | 4693 | 44 | | Hypothetical protein | | DAE48299.1 | | 5e-23 (97.67%) | | 100 | | Siphoviridae sp. | |  |
| Gp44c | 27943:30141 reverse | 82547 | 733 | | DNA polymerase I | | DAG81140.1 | | 0.0 (98.63%) | | 100 | | Siphoviridae sp. | |  |
| Gp45c | 30134:30607 reverse | 18396 | 158 | | HNH endonuclease | | AQN31995.1 | | 2e-115 (98.73%) | | 100 | | *Escherichia* phage P AB-2017 | |  |
| Gp46c | 30662:31288 reverse | 23380 | 209 | | DNA helix destabilizing protein | | DAG23386.1 | | 3e-153 (99.52%) | | 100 | | Siphoviridae sp. | |  |
| Gp47c | 31367:31603 reverse | 9268 | 79 | | Hypothetical protein | | DAN86869.1 | | 5e-23 (62.82%) | | 100 | | Siphoviridae sp. | |  |
| Gp48c | 31600:32841 reverse | 45988 | 414 | | Exonuclease | | QEG06916.1 | | 0.0 (92%) | | 100 | | *Escherichia* coli | |  |
| Gp49c | 32914:33336 reverse | 16259 | 141 | | HNH endonuclease | | QEA10377.1 | | 4e-47 (51.13%) | | 95 | | *Salmonella* phage Shemara | |  |
| Gp50c | 33333:33605 reverse | 10851 | 91 | | Hypothetical protein pink_64 | | QIN98017.1 | | 3e-62 (96.67%) | | 100 | | *Salmonella* phage pink | |  |
| Gp51 | 33649:34146 reverse | 18611 | 166 | | Hypothetical protein vBEcoSRo145clw_00035 | | AUX83710.1 | | 7e-82 (88.55%) | | 100 | | *Escherichia* phage vB_EcoS-Ro145clw | |  |
| Gp52c | 34271:34486 forward | 7759 | 71 | | Transcriptional repressor | | YP_009620128.1 | | 2e-43 (98.59%) | | 100 | | *Escherichia* phage VB_EcoS-Golestan | |  |
| Gp53c | 36711:34504 reverse | 82485 | 736 | | Replicative DNA helicase | | DAQ25533.1 | | 0.0 (97.70%) | | 100 | | Siphoviridae sp. | |  |
| Gp54c | 36708:36836 reverse | 5040 | 43 | | Hypothetical protein G_51 | | AQN31808.1 | | 3e-23 (100.00%) | | 100 | | *Escherichia* phage G AB-2017 | |  |
| Gp55c | 36906:37196 reverse | 11186 | 97 | | Hypothetical protein GT372_00012 | | QIG59279.1 | | 1e-61 (93.75%) | | 100 | | *Escherichia* phage vB_EcoS_XY2 | |  |
| Gp56 | 37193:37375 reverse | 7095 | 61 | | Helix-turn-helix domain protein | | YP_009620128.1 | | 5e-40 (100.00%) | | 100 | | *Escherichia* phage VB_EcoS-Golestan | |  |
| Gp57 | 37849:38040 forward | 7313 | 65 | | Hypothetical protein GT371_00053 | | QIG59244.1 | | 3e-36 (93.44 ) | | 95 | | *Escherichia* phage vB_EcoS_XY1 | |  |
| Gp58 | 38043:38282 forward | 8501 | 80 | | Hypothetical protein | | DAQ72314.1 | | 5e-48 (93.67%) | | 100 | | Siphoviridae sp. | |  |
| Gp59 | 38316:38459 forward | 5540 | 48 | | Hypothetical protein | | DAV78910.1 | | 1e-27 (100.00%) | | 100 | | Siphoviridae sp. | |  |
| Gp60 | 38463:38648 forward | 7077 | 62 | | Protein of unknown function (DUF551) | | DAO31693.1 | | 6e-39 (96.72%) | | 100 | | Siphoviridae sp. | |  |
| Gp61 | 38783:38953 forward | 6871 | 57 | | Hypothetical protein GT371_00048 | | QIG59239.1 | | 5e-32 (94.64%) | | 100 | | *Escherichia* phage vB_EcoS_XY1 | |  |
| Gp62 | 39021:39158 forward | 4843 | 46 | | Hypothetical protein PE1_059 | | QNO11846.1 | | 6e-23 (93.33%) | | 100 | | *Escherichia* phage vB_EcoS_fPoEco01 | |  |
| Gp63 | 39151:39630 forward | 18359 | 160 | | hypothetical protein Schulenburg_066 | | QEG06854.1 | | 1e-94 (84.91%) | | 100 | | *Escherichia* phage Schulenburg | |  |
| Gp64 | 39627:39830 forward | 7410 | 68 | | Hypothetical protein | | DAO35438.1 | | 1e-44 (100.00%) | | 100 | | Siphoviridae sp. | |  |
| Gp65 | 39833:40036 forward | 7859 | 68 | | Hypothetical protein FE2_063 | | QNO11624.1 | | 3e-43 (95.52%) | | 100 | | *Escherichia* phage vB_EcoS_fFiEco02 | |  |
| Gp66 | 40036:40230 forward | 7673 | 65 | | Protein of unknown function (DUF2480) | | DAQ25591.1 | | 1e-38 (98.44%) | | 100 | | Siphoviridae sp. | |  |
| Gp67 | 40227:40520 forward | 11328 | 98 | | Hypothetical protein | | DAG75814.1 | | 1e-63 (93.81%) | | 100 | | Siphoviridae sp. | |  |
| Gp68 | 40596:40904 forward | 10650 | 103 | | Putative holin-like, class II | | YP_009168828.1 | | 8e-56 (96%) | | 100 | | Siphoviridae sp. | |  |
| Gp69 | 40897:41169 forward | 10044 | 91 | | Class I holin-like protein | | YP_009821893.1 | | 4e-61 (98%) | | 100 | | Siphoviridae sp. | |  |
| Gp70 | 41147:41635 forward | 17097 | 163 | | Endolysin | | QNO11705.1 | | 5e-114 (98.15%) | | 100 | | *Escherichia* phage vB_EcoS_fFiEco03 | |  |
| Gp71 | 41816:41965 forward | 5258 | 50 | | Hypothetical protein TE3_066 | | QNO12003.1 | | 5e-25 (91.84%) | | 100 | | *Escherichia* phage vB_EcoS_fTaEco03 | |  |
| Gp72 | 41962:42120 forward | 6128 | 53 | | Protein of unknown function (DUF2737) | | DAG80641.1 | | 3e-32 (100.00%) | | 100 | | Siphoviridae sp. | |  |
| Gp73 | 42117:42353 forward | 8908 | 79 | | Hypothetical protein | | DAQ25598.1 | | 6e-51 (97.44%) | | 100 | | Siphoviridae sp. | |  |
| Gp74 | 42353:42592 forward | 8831 | 80 | | Hypothetical protein | | DAQ25600.1 | | 1e-50 (96.20%) | | 100 | | Siphoviridae sp. | |  |
| Gp75 | 42597:42761 forward | 6664 | 61 | | Hypothetical protein HSE2_gp027 | | AUE23497.1 | | 2e-38 (98.33%) | | 100 | | *Escherichia* phage vB_EcoS_HSE2 | |  |
| Gp76 | 42849:43019 forward | 6676 | 57 | | Hypothetical protein P_34 | | AQN31972.1 | | 7e-37(100.00%) | | 100 | | *Escherichia* phage P AB-2017 | |  |
| Gp77 | 43016:43252 forward | 9049 | 79 | | NinH protein | | QQV88102.1 | | 9e-51 (97.44%) | | 100 | | *Escherichia* phage phiWAO78-1 | |  |
| Gp78 | 43249:43404 forward | 5696 | 52 | | Hypothetical protein FE3_076 | | QNO11714.1 | | 5e-27 (96.08%) | | 100 | | *Escherichia* phage vB_EcoS_fFiEco03 | |  |

**Supplementary Table S2.** Predicted promoters and terminators in the *Escherichia* phage fBC-Eco01 genome

| Promoter or terminator | Sequence | genomic location | Direction |
| --- | --- | --- | --- |
|  |  |  |  |
| Phage RNAP promoter |  |  |  |
| PP1 | TGAATAGTACCCTATTATAT | 3917..3936 | Reverse |
| PP2 | TGAATAGTACCCTATTATAT | 6061..6080 | Reverse |
| PP3 | TGAATAGTATCCTATTATGT | 15129..15148 | Reverse |
| PP4 | ATAATAGTTTGCTATTATGT | 27124..27143 | Reverse |
| PP5 | TGAATAGTACCATATTAATT | 34223..34242 | Forward |
| PP6 | TGAATAGTATCCTATTATGT | 37807..37826 | Forward |
| PP7 | TGAATAGTAGTCTATTCTAT | 38973..38992 | Forward |
| Consensus sequence | tgAATAGTa-ccTATTat-T |  |  |
|  |  |  |  |
| Host RNAP promoter | Sequence (-35 and -10 box) |  |  |
| HP1 | TTGACTTTTCACCAAAAATGTGCTAAGCT | 6159..6187 | Forward |
| HP2 | GTTTACTGAGATTACGCCGTTTCTATGTT | 6320..6348 | Forward |
| HP3 | TTGAAAGTTCTTGACACATGTTTGTATAAT | 37473..37502 | Forward |
| HP4 | TTGACACACAGGAATGTGAACATGACTATCGT | 39542..39573 | Forward |
| HP5 | TTGACACCTAAGATTAATCCGACGATACT | 41632..41660 | Forward |
| HP6 | TTGACTATTATCTATGACACTATTAT | 41754..41779 | Forward |
|  |  |  |  |
| Rho-independent terminators (score) | Sequence, hairpin stem in blue and loop in red | | |
| T1 (-13.10) | AATAAAACTAAGGCCCCGTAGTGGGGCCTTTTTCTTTACT | 3336..3375 | Reverse |
| T2 (-13.40) | GTAAAGAAAAAGGCCCCACTACGGGGCCTTAGTTTTATTT | 3337..3382 | Forward |
| T3 (-12.60) | AAATCGGTAAGGGGCCGATGCCCCCGGCCCaTTTTGGGTGCCG | 4684..4726 | Forward |
| T4 (-9.40) | CATCCCCGCCGCCGGTAGCGCAATACCGGTTTTATCTGCAT | 5172..5212 | Forward |
| T5 (-10.00) | ATAAAAATAAAGCCCCTTAACGGGGCcTTTTATCATTCT | 5491..5529 | Reverse |
| T6 (-10.90) | AATGATAAAAGGCCCCGTTAAGGGGCTTTATTTTTATA | 5493..5530 | Forward |
| T7 (-17.20) | TAAGGACTAGCGCCCCGCTTTAAGGCGGGGCTTTACTTATCGA | 6356..6398 | Forward |
| T8 (-13.00) | GATATAATCGAGAGGGACTTCGGTCCCTCTTTTCATTTACT | 8749..8789 | Forward |
| T9 (-12.20) | TTTCCTTATTACGGCCCCGAAGGGCCGTTGTATTAAGAC | 9335..9373 | Forward |
| T10 (-4.70) | GTATCGGTTGACGTCGGCGCGTCGATGTTTTCAAGCGAG | 11613..11651 | Forward |
| T11 (-13.40) | AATAAGAAAAAGGCCCCTTTCGGGGCCTTAGTTTTATGC | 13113..13151 | Reverse |
| T12 (-14.30) | CATAAAACTAAGGCCCCGAAAGGGGCCTTTTTCTTATTC | 13114..13152 | Forward |
| T13 (-12.00) | GGTAAAGTAAAGCCCTCTTATGAGGGCTTAGTTATTTGT | 25310..25348 | Reverse |
| T14 (-11.40) | CAAATAACTAAGCCCTCATAAGAGGGCTTTACTTTACCT | 25311..25349 | Forward |
| T15 (-12.00) | AATTTAACTAAGGCCCATCATTGGGCCTTTTTACCGGAG | 30613..30651 | Reverse |
| T16 (-10.00) | CGTTTGGTGAAGGGGCTTAACGCCCCTTTCTTTTTACC | 34467..34504 | Forward |
| T17 (-14.30) | GTAAAAAGAAACCCCAACGCCAGAACGTTGGGGTTAAAGTACAAAT | 36864..36909 | Reverse |
| T18 (-14.30) | TTGTACTTTAACCCCAACGTTCTGGCGTTGGGGTTTCTTTTTACC | 36866..36910 | Forward |
| T19 (-17.40) | TAACGATTATTGGGCAGGCTTAAGGCCTGCCCTTTAGTTACCAA | 37691..37734 | Forward |
| T20 (-14.60) | AAAGTCTATATCCGGTCCGGTCGACGGACTGGTATTTCTTCTCA | 40679..40722 | Forward |
| T21 (-12.80) | CTCCAGTTTTATCCCGGCCCTGACCCAGCCGGGATTTTTTTTATCT | 41690..41735 | Forward |
| T22 (-9.80) | TGCCGGTTTGTCGGCATGTCTCAGCCATCGTGCCGTATTCTTTAATC | 43039..43085 | Forward |
| T23 (-12.70) | TCACACCACAAGCCCTCTACGGAGGGCTTTTCTGTACAT | 43405..43443 | Forward |

**Supplementary Table S3.** Genomic similarity (SG) of phage of fBC-Eco01 with related to Caudoviricetes genomes selected to construct VipTree, based on highest similarities standards and genomic similarities (SG>0.2)

| **ID** | **Length** | **Taxid** | **Name** | **Group** | **Host Group** | **Score** | **SG** | **Mean Identity** |
| --- | --- | --- | --- | --- | --- | --- | --- | --- |
| **NC_042084** | 44829 | 2047801 | ***Escherichia* phage VB-EcoS-Golestan** | dsDNA | Gammaproteobacteria | 19685.5 | **0.6677** | **86.2** |
| **NC_041898** | 42765 | 698489 | ***Escherichia* phage K1ind2** | dsDNA | Gammaproteobacteria | 18645.5 | **0.6324** | **85.2** |
| **GU196281** | 43461 | 698490 | ***Escherichia* phage K1ind3** | dsDNA | Gammaproteobacteria | 18768 | **0.6366** | **85.4** |
| **NC_027993** | 43587 | 698486 | ***Escherichia* phage K1G** | dsDNA | Gammaproteobacteria | 18177 | **0.6165** | **84.2** |
| **NC_041897** | 42292 | 698488 | ***Escherichia* phage K1ind1** | dsDNA | Gammaproteobacteria | 18052.5 | **0.6123** | **84.9** |
| **NC_048181** | 44851 | 2565500 | ***Raoultella* phage RP180** | dsDNA | Gammaproteobacteria | 18243.5 | **0.6188** | **85.2** |
| **NC_031925** | 43788 | 1857099 | ***Salmonella* phage BPS11Q3** | dsDNA | Gammaproteobacteria | 15909.5 | **0.5396** | **75.6** |
| **NC_041992** | 42633 | 1204541 | ***Salmonella* phage wksl3** | dsDNA | Gammaproteobacteria | 15578 | **0.5284** | **74.9** |
| **NC_026017** | 41880 | 1540823 | ***Salmonella* phage LSPA1** | dsDNA | Gammaproteobacteria | 16556 | **0.5615** | **79.5** |
| **NC_022752** | 42665 | 424949 | ***Salmonella* phage SETP13** | dsDNA | Gammaproteobacteria | 15809 | **0.5362** | **76** |
| **NC_016763** | 43221 | 1115478 | **Salmonella phage SE2** | dsDNA | Gammaproteobacteria | 15490 | **0.5254** | **74.8** |
| **NC_042065** | 41873 | 1173752 | ***Salmonella* phage FSL SP-101** | dsDNA | Gammaproteobacteria | 15832.5 | **0.537** | **76.9** |
| **NC_022754** | 42749 | 424947 | ***Salmonella* phage SETP7** | dsDNA | Gammaproteobacteria | 15609.5 | **0.5294** | **75.2** |
| **NC_031021** | 41224 | 1868170 | ***Salmonella* phage MA12** | dsDNA | Gammaproteobacteria | 15410 | **0.5227** | **76** |
| **NC_028698** | 41868 | 1611545 | ***Salmonella* phage f18SE** | dsDNA | Gammaproteobacteria | 15430 | **0.5234** | **76** |
| **NC_027994** | 41632 | 698487 | ***Escherichia* phage K1H** | dsDNA | Gammaproteobacteria | 16813.5 | **0.5703** | **83.7** |
| **NC_023608** | 42093 | 1465618 | ***Salmonella* phage vB_SenS-Ent2** | dsDNA | Gammaproteobacteria | 15115 | **0.5127** | **74.7** |
| **NC_024204** | 42764 | 1465613 | ***Salmonella* phage vB_SenS-Ent3** | dsDNA | Gammaproteobacteria | 15050 | **0.5105** | **74.8** |
| **NC_019539** | 42391 | 1163482 | ***Salmonella* phage Ent1** | dsDNA | Gammaproteobacteria | 15021 | **0.5095** | **74.7** |
| **NC_021777** | 43447 | 1340534 | ***Salmonella* phage Jersey** | dsDNA | Gammaproteobacteria | 15276.5 | **0.5181** | **76.9** |
| **NC_009232** | 42572 | 424944 | ***Salmonella* phage SETP3** | dsDNA | Gammaproteobacteria | 14308.5 | **0.4853** | **72.9** |
| **NC_006940** | 40793 | 293644 | ***Salmonella* phage SS3e** | dsDNA | Gammaproteobacteria | 14833 | **0.5031** | **75.3** |
| **NC_041991** | 41546 | 1211279 | ***Salmonella* phage vB_SenS_AG11** | dsDNA | Gammaproteobacteria | 14205 | **0.4818** | **74.4** |
| **NC_021775** | 42215 | 1173749 | ***Salmonella* phage FSL SP-031** | dsDNA | Gammaproteobacteria | 10556 | **0.358** | **65.8** |
| **NC_028695** | 40491 | 1701257 | ***Enterobacter* phage phiEap-2** | dsDNA | Gammaproteobacteria | 10135 | **0.3438** | **63.8** |
| **NC_021317** | 21248 | 1325963 | ***Salmonella* phage L13** | dsDNA | Gammaproteobacteria | 8728 | **0.296** | **76** |
| **NC_021563** | 42724 | 1282995 | ***Serratia* phage Eta** | dsDNA | Gammaproteobacteria | 5872 | **0.1992** | **59.8** |
| **NC_025466** | 40161 | 1458863 | ***Shewanella* sp. phage 3/49** | dsDNA | - | 4047.5 | **0.1373** | **53.1** |
| **NC_016566** | 44077 | 1109721 | ***Shigella* phage EP23** | dsDNA | Gammaproteobacteria | 4193 | **0.1422** | **56.4** |
| **NC_013600** | 45169 | 673375 | ***Sodalis* phage SO1** | dsDNA | Gammaproteobacteria | 4160 | **0.1411** | **56.6** |
| **NC_019724** | 43741 | 1147142 | ***Escherichia* phage HK578** | dsDNA | Gammaproteobacteria | 4067.5 | **0.138** | **57.1** |
| **NC_031113** | 44513 | 1883202 | ***Escherichia* phage Gluttony** | dsDNA | Gammaproteobacteria | 4013 | **0.1361** | **56.7** |
| **NC_024783** | 44332 | 1527514 | ***Escherichia* phage EK99P-1** | dsDNA | Gammaproteobacteria | 4062 | **0.1378** | **57.4** |
| **NC_028901** | 43900 | 1720498 | ***Escherichia* phage slur05** | dsDNA | Gammaproteobacteria | 3982.5 | **0.1351** | **57.1** |
| **NC_031098** | 40749 | 1852629 | ***Acinetobacter* phage vB_AbaS_TRS1** | dsDNA | Gammaproteobacteria | 946.5 | **0.0321** | **40** |
| **NC_028809** | 58637 | 1175663 | ***Pseudomonas* phage PaMx74** | dsDNA | Gammaproteobacteria | 829 | **0.0281** | **42** |
| **NC_054638** | 45126 | 2591136 | ***Salmonella* phage vB_SenS_SB28** | dsDNA | Gammaproteobacteria | 751.5 | **0.0255** | **44.9** |
| **NC_028931** | 55108 | 1175659 | ***Pseudomonas* phage PaMx28** | dsDNA | Gammaproteobacteria | 659 | **0.0224** | **43.6** |
| **NC_013644** | 37856 | 673839 | ***Enterococcus* phage phiFL4A** | dsDNA | Firmicutes | 711.5 | **0.0241** | **40.7** |
| **MN995824** | 37561 | 2712942 | ***Enterococcus* phage EFP1** | dsDNA | Firmicutes | 714 | **0.0242** | **40.7** |
| **NC_048790** | 45644 | 2588502 | ***Corynebacterium* phage Lederberg** | dsDNA | Actinobacteria | 681 | **0.0231** | **42.8** |
| **NC_048787** | 44381 | 2588501 | ***Corynebacterium* phage Dina** | dsDNA | Actinobacteria | 680 | **0.0231** | **42.8** |
| **NC_025471** | 36844 | 1527515 | ***Idiomarinaceae* phage Phi1M2-2** | dsDNA | Gammaproteobacteria | 605 | **0.0205** | **55.3** |
| **NC_054636** | 46454 | 2282196 | ***Shigella* phage Sf11 SMD-2017** | dsDNA | Gammaproteobacteria | 681 | **0.0231** | **50.2** |
| **NC_054647** | 45367 | 2562461 | ***Salmonella* phage Akira** | dsDNA | Gammaproteobacteria | 632.5 | **0.0215** | **45.9** |
| **NC_054644** | 46905 | 2301723 | ***Salmonella* virus VSt472** | dsDNA | Gammaproteobacteria | 604.5 | **0.0205** | **45.6** |
| **NC_054641** | 48608 | 2583286 | ***Salmonella* virus KFS-SE2** | dsDNA | Gammaproteobacteria | 736.5 | **0.025** | **45.6** |

**Supplementary Table S4.** Heatmap showing pairwise intergenomic distances and similarities (the nucleotide and amino acid identities) among whole genome phage of fBC-ECO01 and 25 homologs selected Caudoviricetes based on highest similarities standards and genomic similarities (SG>0.2)

a The group indicated in green represent the jersey genus

b The group indicated in yellow represents the ***Kagunavirus*** genus


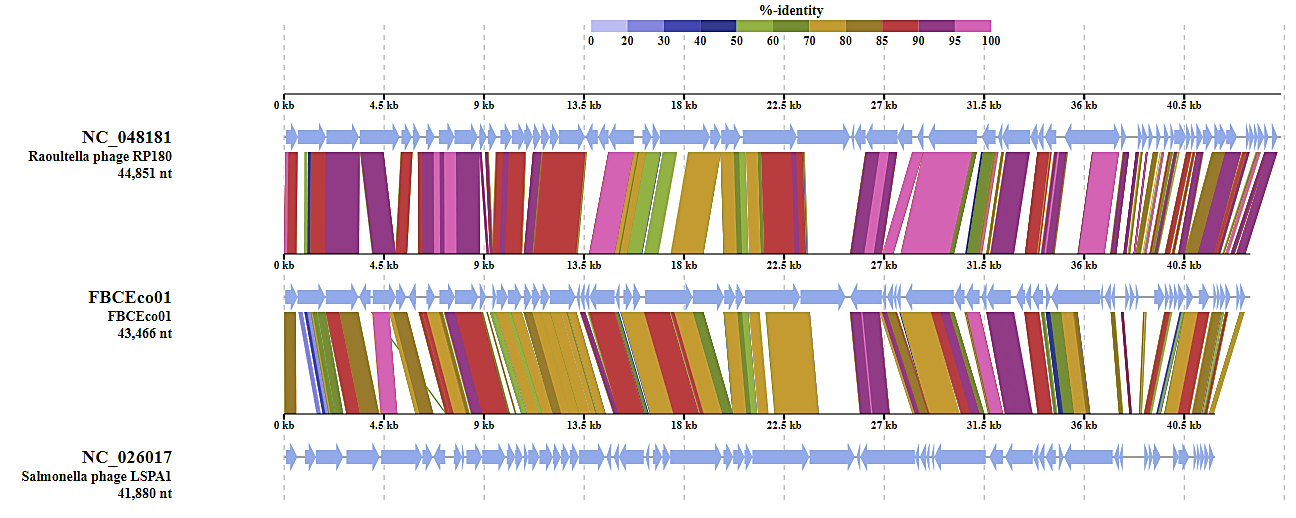


**Supplementary Figure S1.** Representation of a genomic alignment of phage fBC-Eco01 with *Salmonella* phage LPS1 and *Raoultella* phage RP180 generated by TBLASTX using ViPTree server (Nishimura Y, Yoshida T, Kuronishi M, Uehara H, Ogata H, Goto S. 2017. ViPTree: the viral proteomic tree server. Bioinformatics 33:2379-2380). Homologous regions are connected by colored fragments based on amino acid identity. The color bar indicates identity percentages according to TBLASTX
